# SnO_2_/MWCNTs Nanostructured Material for High-Performance Acetone and Ethanol Gas Sensors

**DOI:** 10.1021/acsomega.4c10981

**Published:** 2025-02-12

**Authors:** Mikayel Aleksanyan, Artak Sayunts, Gevorg Shahkhatuni, Zarine Simonyan, Davit Kananov, Emma Khachaturyan, Rima Papovyan, Alena Michalcová, Dušan Kopecký

**Affiliations:** † Center of Semiconductor Devices and Nanotechnologies, Yerevan State University, 1 Alex Manoogian, 0025 Yerevan, Armenia; ‡ Department of Metals and Corrosion Engineering, University of Chemistry and Technology Prague, Technická 5, Prague 6, Prague 166 28, Czech Republic; § Department of Mathematics, Informatics and Cybernetics, Faculty of Chemical Engineering, University of Chemistry and Technology Prague, Technická 5, Prague 6, Prague 166 28, Czech Republic

## Abstract

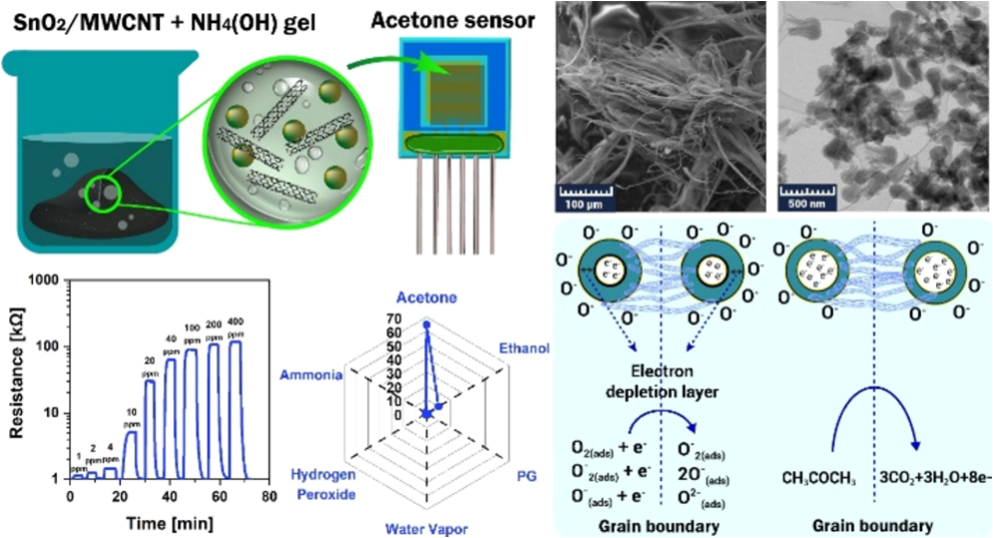

This work presents a novel nanostructured material SnO_2_/multiwalled carbon nanotubes (MWCNTs) as a sensing film for the detection of acetone and ethanol vapors. The fabrication of SnO_2_/MWCNT chemoresistive sensors demonstrates a cost-effective hydrothermal method using a centrifugation technique. The material investigation of the synthesized SnO_2_/MWCNTs nanocomposite represents various techniques, such as scanning electron microscopy (SEM), transmission electron microscopy (TEM), energy-dispersive X-ray (EDX) elementary analysis, EDX mapping, and X-ray diffraction (XRD) analysis. The SnO_2_/MWCNTs sensor exhibits rapid response/recovery behavior toward acetone (53/5 s) and ethanol (86/3 s) while showing satisfactory values of responsiveness (*S*_act_ = 90.5 and *S*_etn_ = 21, *n* = 100 ppm). The low detection limit of these vapors is assigned a concentration of 1 ppm, where discernible responses are elicited. Thus, the SnO_2_/MWCNTs sensor production efforts have yielded a high-end volatile organic compound (VOC) detector, highly suitable for human technological and engineering activity.

## Introduction

Acetone ((CH_3_)_2_CO) is considered one of the most widely used materials that successfully serve in industry and technology for preparing various dyes, developing chemical processes, cleaning various surfaces, etc.^1−3^ The use of acetone in technological processes in the form of various solvents makes direct human contact with this substance inevitable, which can lead to serious health problems. Direct contact with acetone can cause headaches, nausea, fatigue, fainting, and damage to the nervous system. It also negatively affects the biological activity of the human liver, kidney, and pancreas.^4,5^ Furthermore, as part of the composition of human exhaled air, acetone serves as a good biomarker of diabetes.^6^ The detection and measurement of acetone vapor concentrations in human exhaled breath is a noninvasive method of diagnosing diabetes. The concentration of acetone in the exhaled air of a healthy person ranges from 0.3 to 0.9 ppm, while it can reach 1.8 ppm in a person with type II diabetic patients.^7,8^ Thus, the accurate identification of ultralow concentrations of acetone vapor by different gas sensors can successfully serve both as a preventive measure for human health risks and as a noninvasive system for the diagnosis of diabetes. Another important representative of volatile organic compounds (VOCs) is ethanol, which is also widely used in various industries and medicine.^9−12^ It serves as a solvent for the synthesis of different organic materials. It is also considered an ecologically clean fuel (gasohol) for various vehicles. Ethanol is best used in medicine as a bacteria-killing agent for various surfaces and human skin. In different alcoholic beverages (wine, beer, etc.), it is considered as an intoxicating ingredient.^13,14^ Thus, the accurate and selective detection of ethanol concentrations is another challenge facing researchers.

Currently, VOCs are mainly detected by combustion, optical, electrochemical, FET-based (field-effect transistor), and semiconductor gas sensors.^15,16^ Although the presented sensor structures have pronounced features for application in the given environment, the most sophisticated are considered semiconductor chemoresistive sensors. These are characterized by a particularly simple structure, easy fabrication technology, low cost, and high sensitivity, which are also compatible with modern micro- and nanoelectronic devices.^17−20^ The nanostructured metal oxide materials used here (SnO_2_, ZnO, In_2_O_3_, Fe_2_O_3_, etc.) have high mechanical, chemical, and temporal stability, simultaneously exhibiting good eco-friendly behavior.^21,22^ Metal oxide-based nanostructures are distinguished by their high sensitivity, rapidity, and stability to VOCs, and their synthesis technologies are quite straightforward. These materials are classified as p-type or n-type behavior semiconductors exhibiting an electrical resistance change as a gas response in the presence of target gases.^23−25^ In particular, SnO_2_ is one of the most widely used metal oxides in semiconductor gas sensors, which has a wide energy band gap (3.7 eV), high melting point (1630 °C), n-type semiconductor behavior and appears mainly with the rutile crystal structure (electron mobility: (70–260) cm^2^/(V × s), molar mass: 150.708 g·mol^–1^, density: 6.95 g/cm^3^ (20 °C), heat capacity: 52.6 J/mol·K, refractive index: 2.006, lattice constant: *a* = 4.737 Å, *c* = 3.185 Å, α = 90°, β = 90°, γ = 90°).^26−32^ Tin oxide is widely used as a supersensitive material for a wide variety of gas sensors that exhibit high temporal stability and speed.^33−35^

In the onerous processes of solving modern nanotechnological issues and developing new types of nanosystems, nontraditional one-dimensional (1D) and two-dimensional (2D) materials come to the rescue, which in recent decades have irreversibly changed the challenges in this field.^36−40^ Carbon-based nanomaterials have gained indescribably great demand and application here, which is justified by their unique electrical, chemical, physical, optical, and mechanical properties.^41,42^ Multiwalled carbon nanotubes (MWCNTs) are considered to be one of the bright representatives of these materials used in gas sensors due to their high surface area, chemical stability, high carrier mobility, good conductivity, well-catalytic properties, and excellent flexibility (Electron mobility: (10^4^–10^5^) cm^2^/(V × s), Young’s modulus: ∼1–1.28 TPa, tensile strength: ∼100 GPa, specific strength: 48,000 kN m/kg, density: 1.3–1.4 g/cm^3^, electrical conductivity: 10^6^ S m^–1^(SWCNT) and 10^5^ S m^–1^ (MWCNT), thermal conductivity: 0.1–6600 Wm^–1^ K^–1^, Raman: G band = ∼1584 cm^–1^, *G*′ band = ∼2600–2800 cm^–1^, D band = ∼1300–1400 cm^–1^).^43−46^ The combination of metal oxides with MWCNTs leads to synergistic effects of the advantages mentioned above, which are successfully applied in modern sensor systems.^47−49^ Tin oxide is responsible for converting chemical reactions with gas molecules into an electrical signal. Here, the introduction of nanotubes leads to an increase in the effective surface area of the sensor, which, in turn, leads to an increase in sensitivity. Nanotubes can presumably serve as dopants in basic metal oxides, bringing their baseline resistance into measurable ranges. Besides, due to the incomparably higher mobility of charge carriers in the CNT,^46,50^ the sensor based on the composite material becomes faster. Thus, the composite combination of these two materials leads to high sensor performance.^51−53^

Herein, a gas-sensitive SnO_2_/MWCNTs nanomaterial was synthesized by a low-cost and simple hydrothermal method, the material characteristics of which were thoroughly investigated by scanning electron microscopy (SEM), transmission electron microscopy (TEM), energy-dispersive X-ray (EDX), and X-ray diffraction (XRD) spectroscopy. Gas-sensing measurements revealed that the MWCNTs/SnO_2_ material was an excellent candidate for the detection of acetone and alcohol vapors. The novelty of the work lies in the fact that acetone and ethanol sensor with high sensitivity, speed, and selectivity was obtained by a low-cost and simple technological method, expressing the trade-off of all key parameters.

## Experimental Section

### Synthesis of the SnO_2_/MWCNTs Nanomaterials

**Figure 1:**
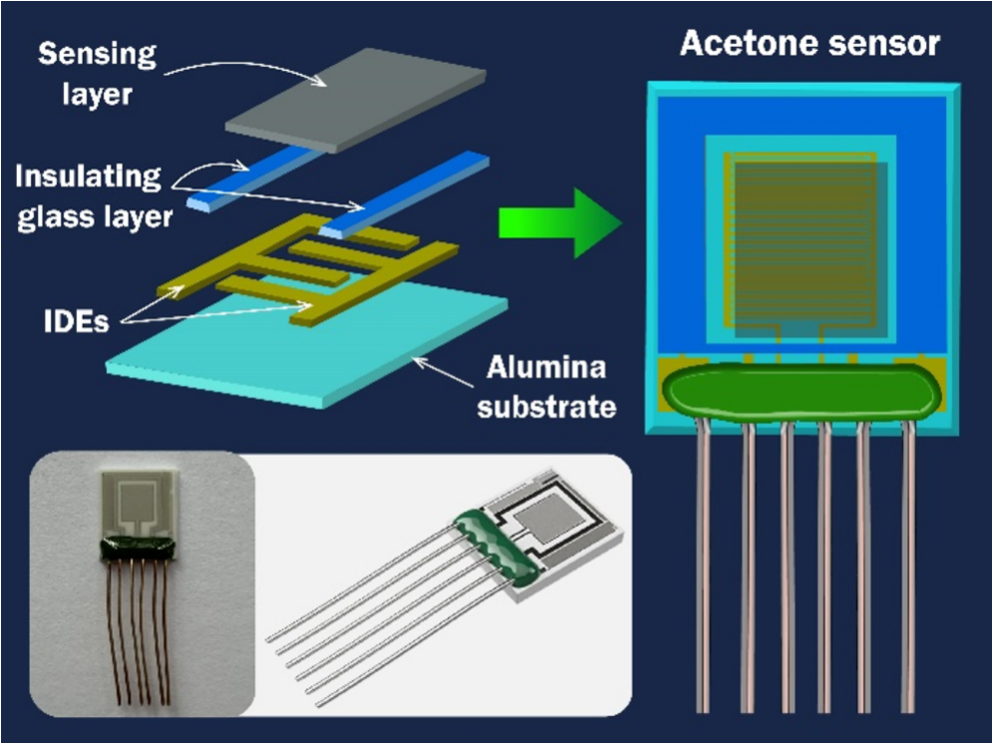
Schematic illustration of the chemoresistive gas sensor structure.

For the fabrication of sensors based on tin oxide coated with MWCNTs (SnO_2_/MWCNT), a cost-effective hydrothermal method was applied.^54^ Initially, the purified MWCNTs were dispersed in distilled water and applied ultrasound for 30 min. Then, a calculated amount of a SnCl_2_·2H_2_O solution was dissolved in distilled water with a small amount of hydrochloric acid. Afterward, both solutions were mixed under ultrasound for 30 min. The precursor was applied to the hydrothermal synthesis by an autoclave for 24 h at 150 °C. The nanocomposite powder was then filtered and dried at 90 °C for 5 h. The result was the MWCNT/SnO_2_ nanocomposite powder, which was finally calcinated at 45 °C for 5 h. Before the suspension was prepared and applied to the dielectric substrate, the resulting powder was finely crushed and mixed in a mortar to achieve homogeneity. The resulting powder was introduced into a 10% NH_4_(OH) aqueous solution (as a binder) to obtain a viscous suspension that was ready for centrifugation. Before obtaining the gas-sensitive film, factory-produced sensor substrates (Figure 1) were subjected to chemical cleaning. The substrates were boiled in distilled isopropanol for 10 min, and surface cleaning was applied using toluene and ethanol solutions.

**Figure 2:**
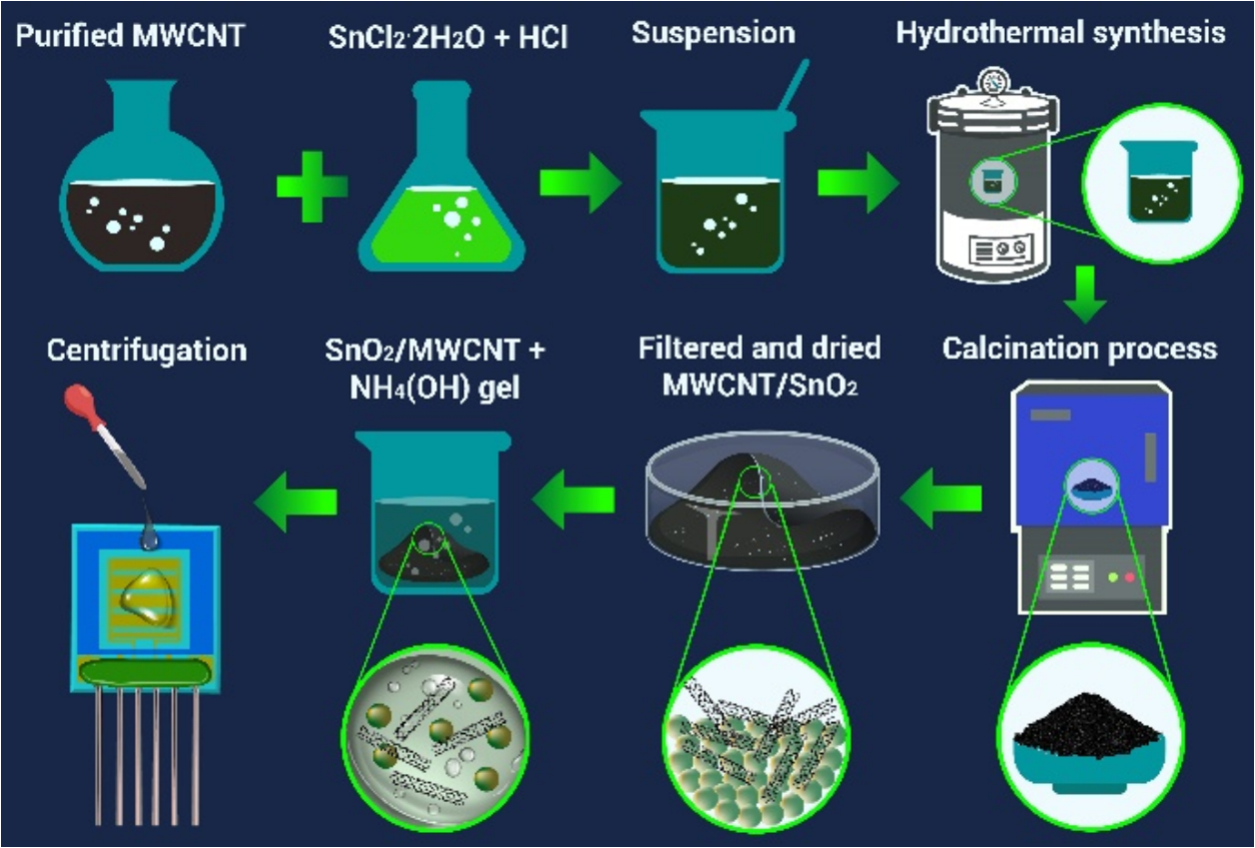
Technological flow of SnO_2_/MWCNT sensor production.

The obtained SnO_2_/MWCNT gel was centrifuged on the substrate using the Laurell WS-650HZB-23NPPB centrifuge machine (1000 rpm −60 s). The film thickness was measured using a Thin Film Thickness Measurement Profiler (D-500), which was in the range of 600–750 nm. To improve film adhesion and eliminate aqueous composition, the fabricated sensors were annealed at 350 °C for 4 h. At higher temperatures (>350 °C), annealing can lead to an increase in the degree of oxidation of the film and make structural changes (formation of agglomerations), which are indicators of a decrease in the gas response. At lower temperatures (<350 °C), the adhesion properties of the film with the substrate are affected, and instability of the gas-sensing parameters is observed during further application. Finally, the sensing surfaces were ruthenized with a 0.03 M Ru(OH)Cl_3_ solution and calcinated at 100 °C for 20 min. For the comparison of gas-sensing parameters, pristine nanomaterials (SnO_2_ and MWCNTs) were also synthesized, and sensors were prepared on the basis of them. The technological flow of sensor production is shown in Figure 2.

The gas-sensing behavior of the SnO_2_/MWCNT sensor was studied by the gas-testing setup.^55^ The well-known method was applied to obtain the concentrations of acetone vapor in the measuring chamber.^56^ The sensor response was defined as a ratio of the film resistances in pure air (*R*_air_) and the acetone vapor environment (*R*_gas_), respectively.

## Results and Discussion

### Characterization

**Figure 3:**
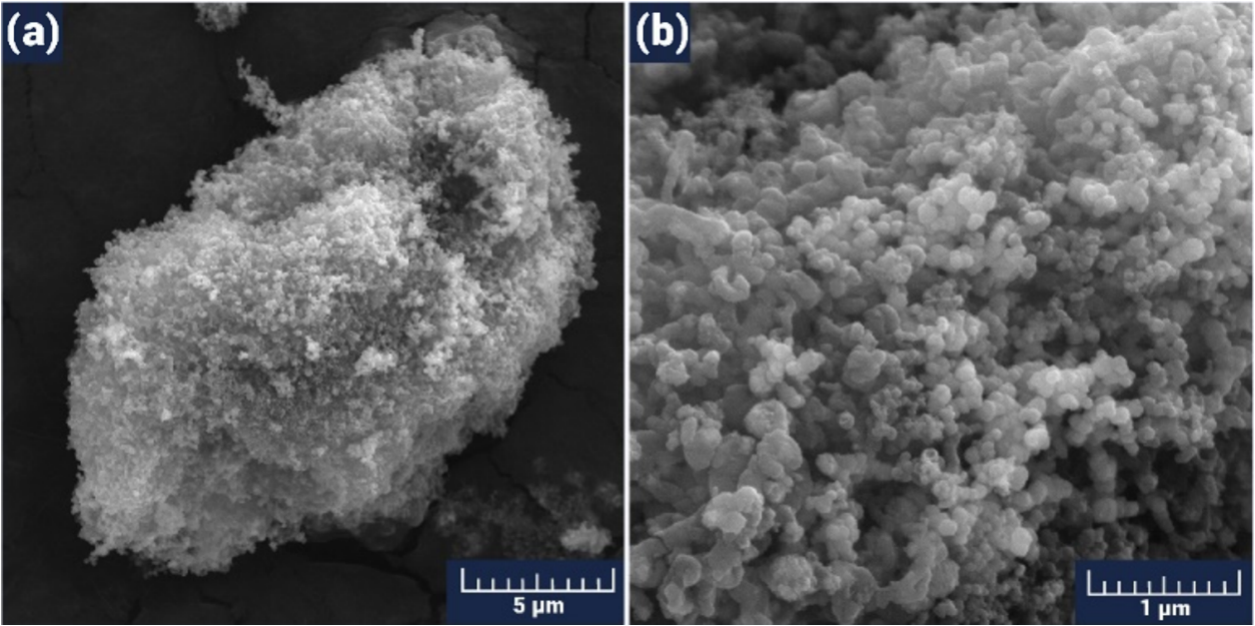
SEM images of the pristine SnO_2_ powder with scale bars of 5 (a) and 1 μm (b).

To better understand the gas-sensing mechanisms and reveal the structural parameters of the used nanomaterials, SEM measurements were carefully performed with a MIRA 3 LMH (Tescan) instrument. First, the SEM characteristics of the pure SnO_2_ powder, which had an obvious nanogranular structure, were investigated (Figure 3).

Here, the grain sizes varied over a wide range (20–300 nm), with the individual grains mainly in contact through the necks. It was assumed that the interconnection process of adjacent grains and the formation of agglomerations (combining several grains) occurred due to chemical treatment and the application of temperature. Here, the number of grains with a small diameter was predominant, which was a favorable condition to obtain a high gas sensitivity.^57^ The use of high temperatures contributes to the formation of agglomerations,^58^ which is why low-temperature processes were used mainly in the synthesis process.

**Figure 4:**
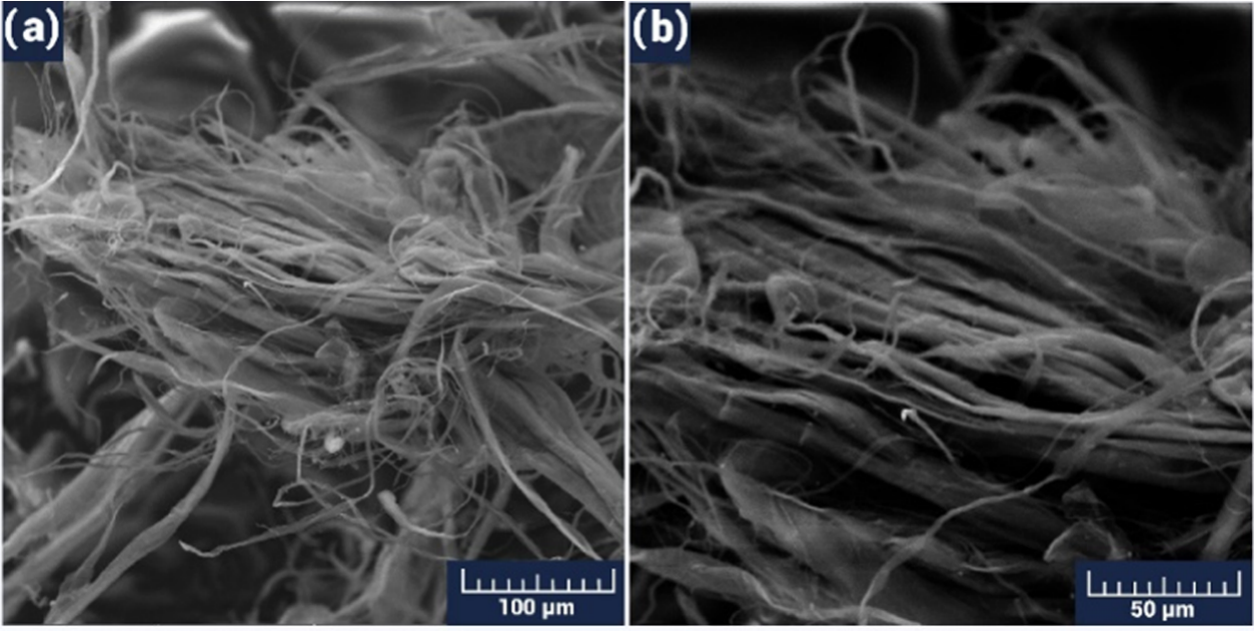
SEM image of the MWCNT material with a scalebar of 100 μm (a) and 50 μm (b).

The structural and crystallographic characteristics of the pristine MWCNTs used in the sensing material were also investigated. In the initial stage of use, the MWCNTs had the appearance of a rather mixed interwoven hair-like structure (Figure 4). Here, we can find nanotubes with a diameter of 100 nm to tens of micrometers, where the large diameter tubes, in turn, consist of smaller ones. Their appearance became more visible as a result of further chemical and mechanical processing.

**Figure 5:**
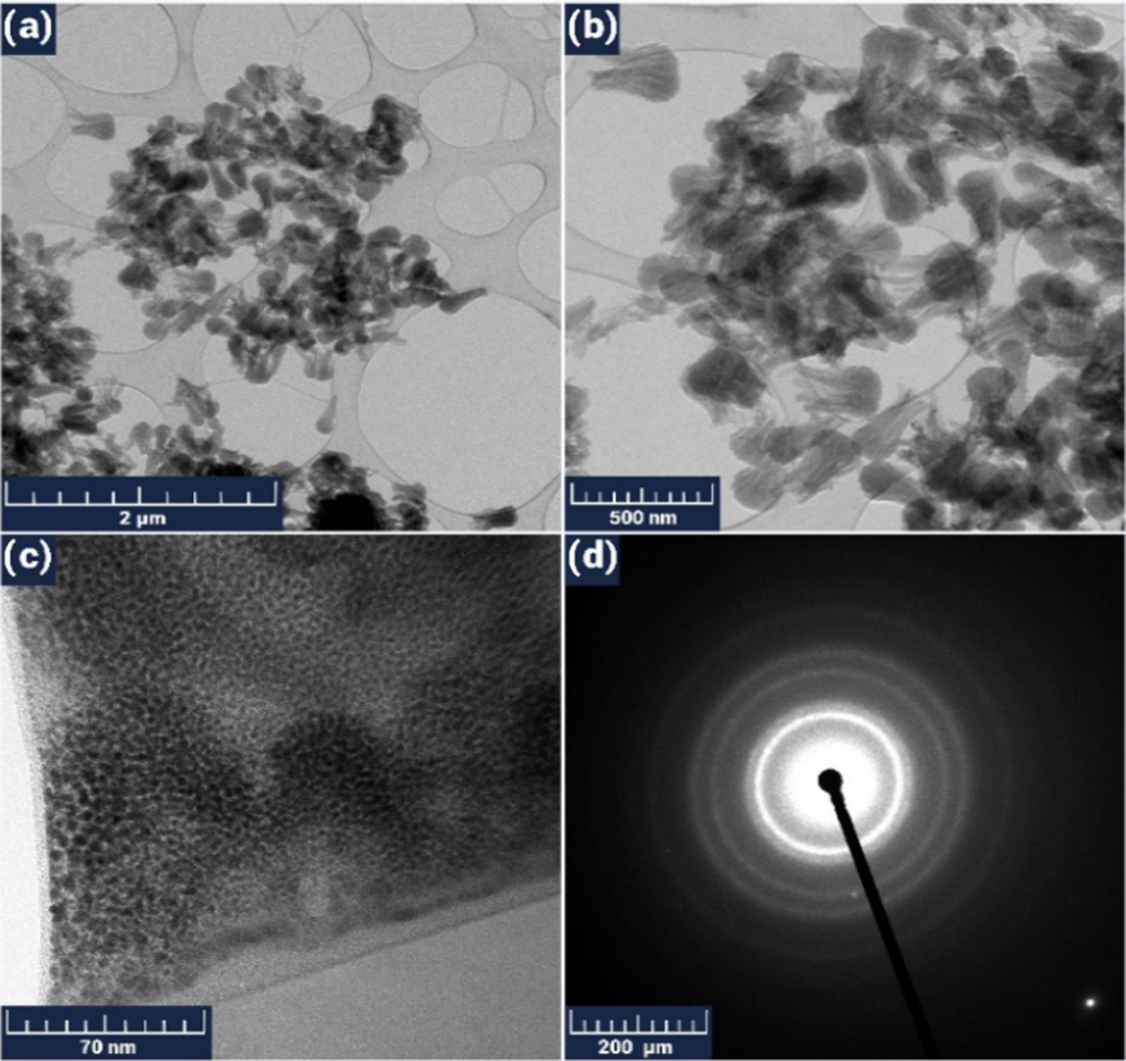
Low (a, b) and high (c) resolution TEM images (with scale bars of 2 μm, 500 nm, and 70 nm, respectively) and SAED pattern (d) for the CNTs.

A TEM technique was applied to the processed and cut nanotubes using a Jeol 2200 FS instrument with 200 kV of accelerating voltage. Here, their tubular appearance and crystal structure were clearly visible, where branching and changing in diameter values along the tubes were also observed (Figure 5).

This type of structure is quite favorable for the effective adsorption and subsequent diffusion of target gas molecules. The gas-sensitive matrix was further improved when pure nanotubes were combined with metal oxide nanoparticles to form a composite nanomaterial.

**Figure 6:**
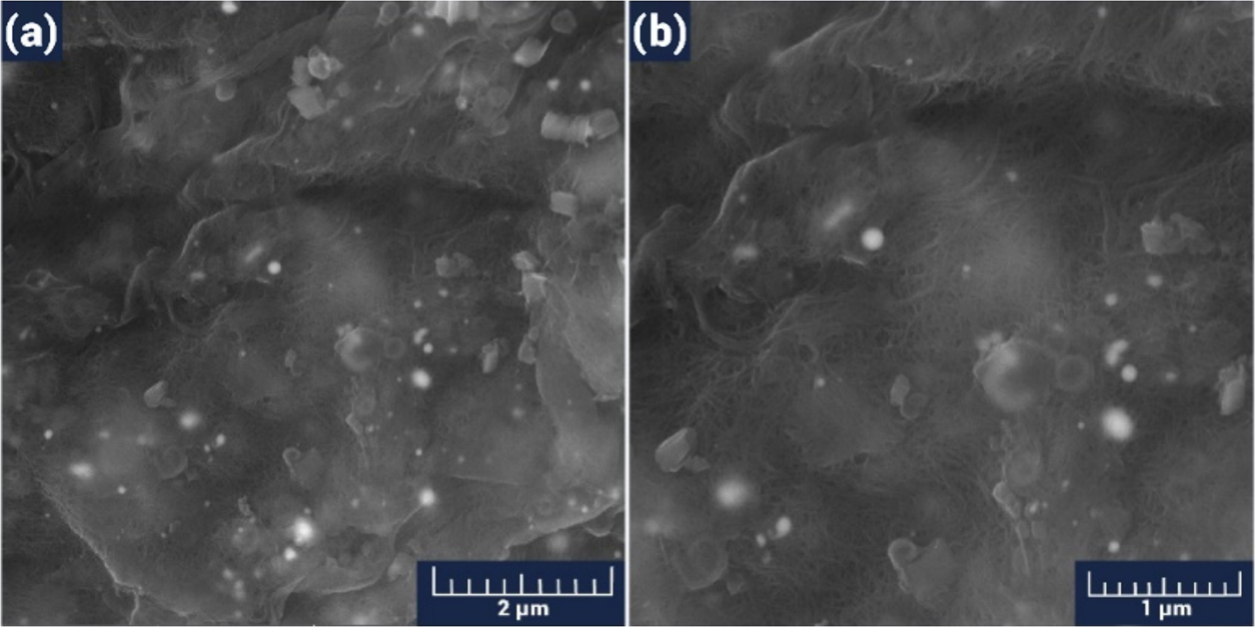
SEM images of the SnO_2_/MWCNTs film with a scalebar of 2 μm (a) and 1 μm (b).

The structural properties of this composite were also investigated by using the SEM technique. In the dense forest of the nanotubes, tin oxide nanoparticles were prominently distributed (Figure 6), which presumably serve as unique centers for driving the chemisorption processes. Carbon nanotubes can also act as unique bridges between metal oxide nanoparticles, controlling the transfer of charge carriers from one grain to another and the thickness of the depletion layer at the near-interface region of the grains.

**Figure 7:**
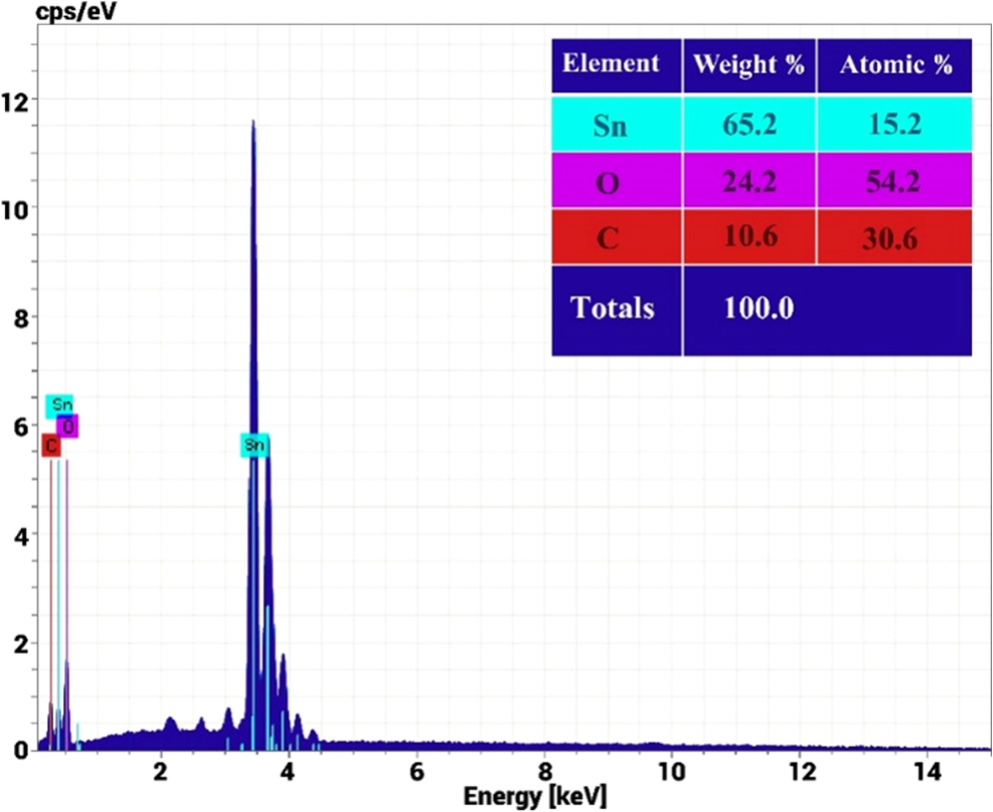
EDX spectrum of the SnO_2_/MWCNTs material.

To extract the actual concentrations of the individual components in the synthesized SnO_2_/MWCNTs material, Quantax 200 energy-dispersive X-ray Spectroscopy (EDX) was used (with the XFlash 6 | 10 detector (Bruker) with a resolution of 127 eV and an acceleration voltage of 15 kV). The carbon composition in the parent material was 10.6 and 30.6% represented by weight and atomic percentages, respectively (Figure 7). Compared to the composition used in our previous work, which contained 15.2 at. % of carbon,^55^ here, we have more than 2 times higher carbon content, which led to a significant deviation of the gas-sensing characteristics of the acetone sensor. The presence of oxygen (24.2 wt %) was mainly determined by the oxygenated composition of tin oxide and the absorption of oxygen species from the atmosphere.

**Figure 8:**
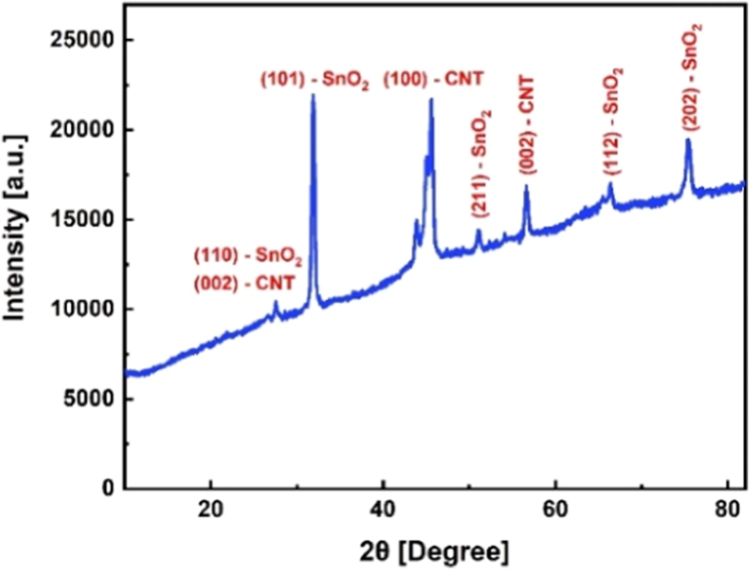
XRD pattern of the SnO_2_/MWCNTs material.

The XRD pattern of the SnO_2_/MWCNTs material is shown in Figure 8, where the peaks of (002) and (100) can be attributed to the availability of the MWCNTs. The diffraction peaks of (110), (101), (211), (112), and (202) were characterized by the tin oxide.^59,60^

**Figure 9:**
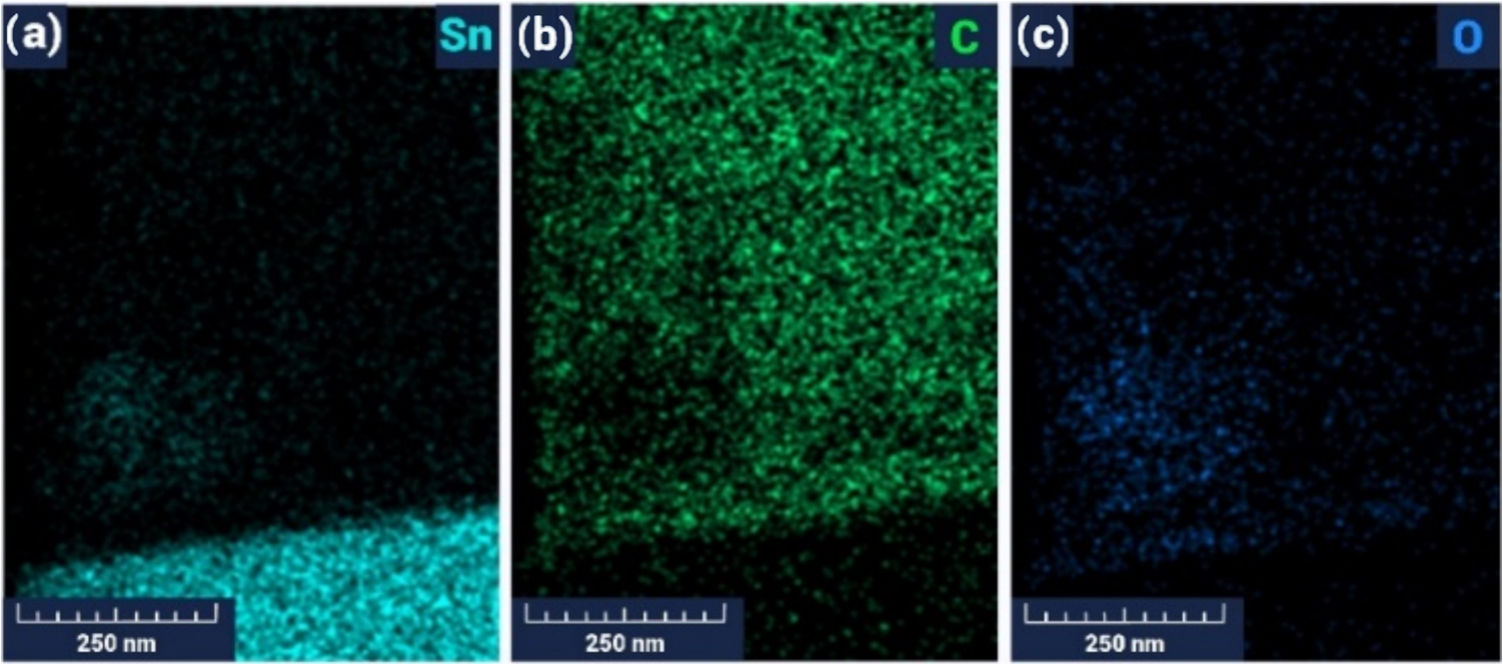
EDX elemental mapping analysis of Sn (a), C (b), and O (c) in the SnO_2_/MWCNTs material.

The surface of the SnO_2_/MWCNTs material was studied by EDX elemental mapping analysis (Oxford Instruments). Figure 9 reflects the evenly distributed elements (Sn, C, and O) in the SnO_2_/MWCNTs material.

### Gas-Sensing Properties

**Figure 10:**
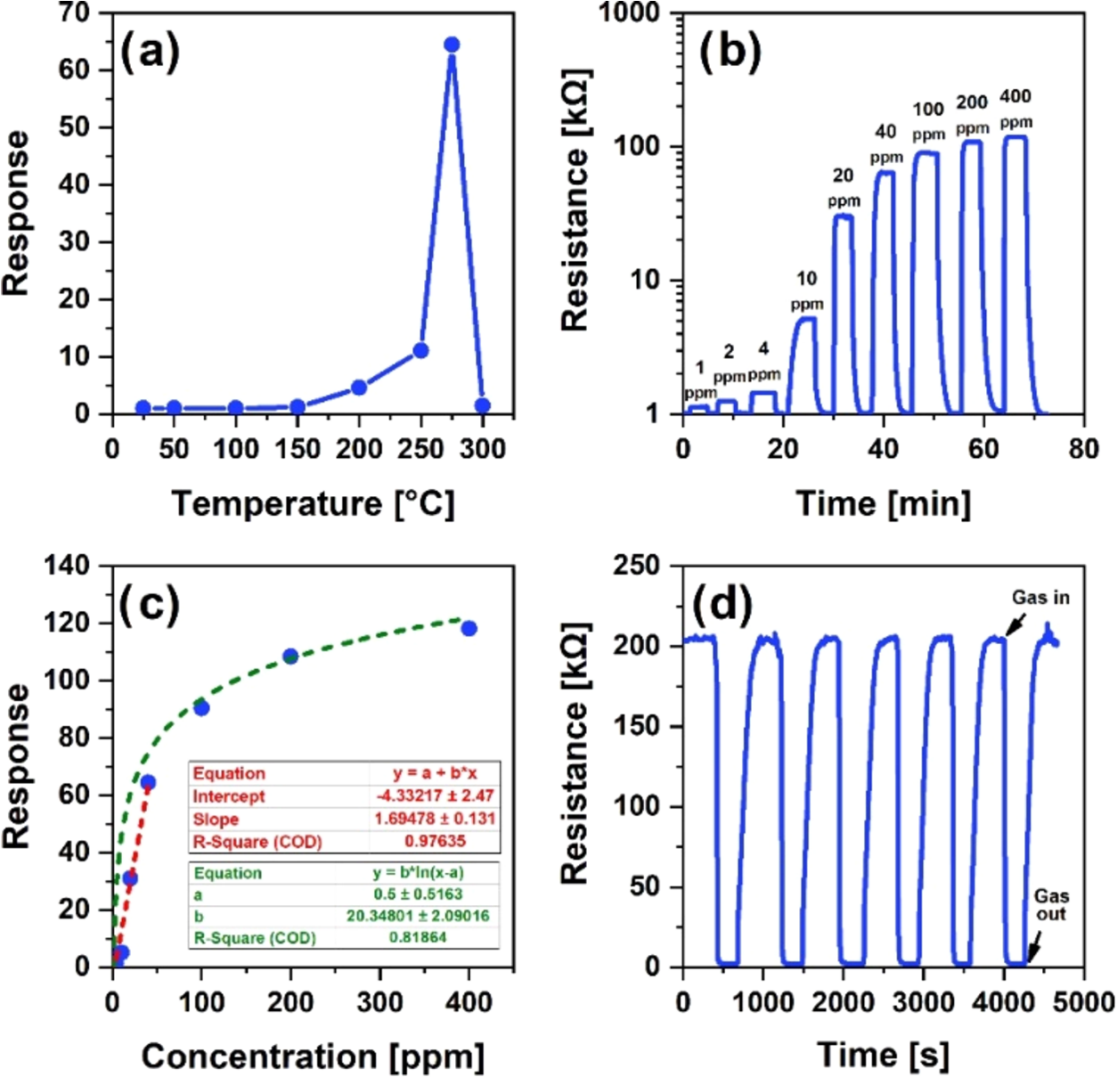
Dependence of the acetone response on the operating temperature in the range of 25–300 °C at 40 ppm acetone concentration (a), acetone responses toward different concentrations at 275 °C (b), dependence of the sensor’s response on acetone vapor concentration at 275 °C (c), and response repeatability of the sensor to 100 ppm acetone vapor expressed in 6 cycles at 275 °C (d).

It is known that the response of the chemoresistive sensors is highly dependent on the temperature of the gas-sensitive film. Chemisorption and diffusion processes, which play a key role in gas-sensing phenomena, require thermal activation energies, leading to a strong thermal dependence on the response.^61,62^ Thus, to choose the optimal working temperature of the sensor, its response to acetone was investigated in detail in the temperature range 25–300 °C. From room temperature to 150 °C, the response of the sensor did not reach high values, which was due to the almost absence of chemisorption processes under cold conditions (Figure 10a). In the temperature range of 150–250 °C, a restrained increase in the response was observed, which was presumably due to the interlocal interaction of oxygen species and gas molecules. Here, a pronounced response peak was observed at 275 °C, which corresponded to the most effective precondition for the gas–semiconductor interaction. At a higher temperature (>275 °C), due to the predominance of the rate of the acetone vapor desorption processes, a sharp decrease in sensitivity was observed. Thus, as the most effective point for performing physicochemical processes, 275 °C was considered as the working temperature for the detection of acetone. At this temperature, the gas-sensing characteristics were carefully considered, extracting data on the response, speed, stability, and other performance parameters of the sensor. In particular, the real response of the sensor was observed in the case of different concentrations of acetone vapors in the range of 1–400 ppm (Figure 10b). The low detection limit of the sensor was recorded at a concentration of 1 ppm acetone, while the higher response values were registered at the next higher levels of acetone trace concentration. Notably, a concentration of 400 ppm was assigned a fairly high response value (118), demonstrating the high performance of the sensor.

Although at higher values in this concentration range, an apparent saturation trend was observed in the response (Figure 10c). For acetone concentrations in the range of 1–50 ppm, the curve can be approximated as linear, in which case the presence of vacancies for the adsorption centers on the active surface was assumed. The sensor’s ability to be used repeatedly and continuously was verified by the degree of repeatability of the response values under the same conditions. Sensitivity measurements were performed in 6 different cycles at 100 ppm, corresponding to response values of 88.57, 89.75, 83.17, 85.54, 90.11, and 88.57 (Figure 10d). These correspond to the average value of 87.62, where the maximum deviation ratio of the average response was attributed to 5%.

**Figure 11:**
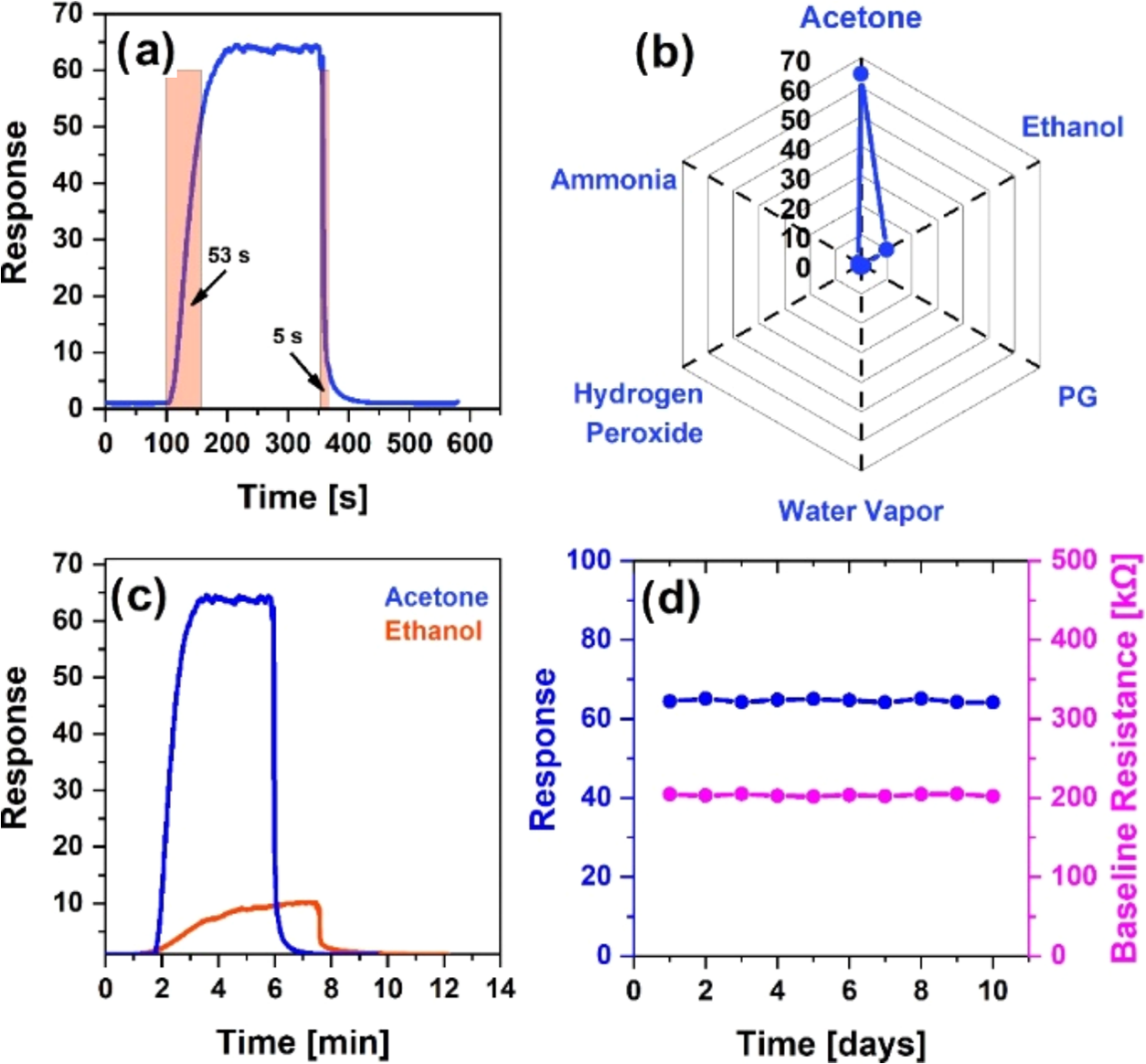
Change in the dynamic response of the sensor reflecting the response and recovery times at 40 ppm of acetone (a), acetone selectivity of the sensor to different environmental gases (b), comparison of sensor responses to 40 ppm of ethanol and acetone vapors at 275 °C (c), stability, and baseline resistance of the sensor in 400 ppm acetone vapor (d).

Resistance saturation times over the dynamic response curve were observed to evaluate the sensor performance. Here, the response and recovery times were veiled by expressing their values of 53 and 5 s, respectively (Figure 11a).

It is quite remarkable that the recovery time is extremely short, which is an indicator of the fast and complete recovery of the sensor. Usually, the recovery processes in chemisorption sensors are quite slow due to the heavy desorption of gas molecules from the sensitive surface.^63^ Here, such a fast behavior can definitely be attributed to the high operating temperatures.^64^

The sensor was also selectively tested for other ambient gases, which highlighted its potential for real-life applications. The response of the SnO_2_/MWCNTs sensor to ethanol (*n* = 40 ppm), propylene glycol (PG) (*n* = 55 ppm), water vapor (*n* = 3300 ppm), hydrogen peroxide (*n* = 225 ppm), and ammonia (*n* = 130 ppm) gases was 10, 1.1, 1.07, 1.08, and 1, respectively (Figure 11b). These results were compared with the response (65) to 40 ppm acetone concentrations, which was several times higher than those for other trace gases, demonstrating fairly high selectivity of the sensor. Among the selectivity results, the sensor’s extreme insensitivity to humidity was also noteworthy. Besides, by changing the concentration of the aqueous acetone solution in the measuring chamber, different humidity levels were established there, but these did not affect the results of the acetone sensing measurements.

At the operating temperature of 275 °C and the same concentration (40 ppm), the sensor’s actual response curves in acetone and ethanol were also compared (Figure 11c). Under these conditions, the sensor was characterized as an acetone detection system in terms of both responsiveness and operating speed.

To confirm the long-term stability of the sensor, the responses of the sensor to 40 ppm acetone vapor were measured under the same physical conditions for 10 days corresponding to the values of 64.5, 65.1, 64.3, 64.8, 65.02, 64.7, 64.2, 65.08, 64.3, and 64.2, respectively (Figure 11d). Besides, the sensor’s baseline resistance did not also change significantly over the days. The maximum deviation from the average response value in the received pattern was quite small (0.74%), which was a direct indicator of the high temporal stability of the sensor response.

**Figure 12:**
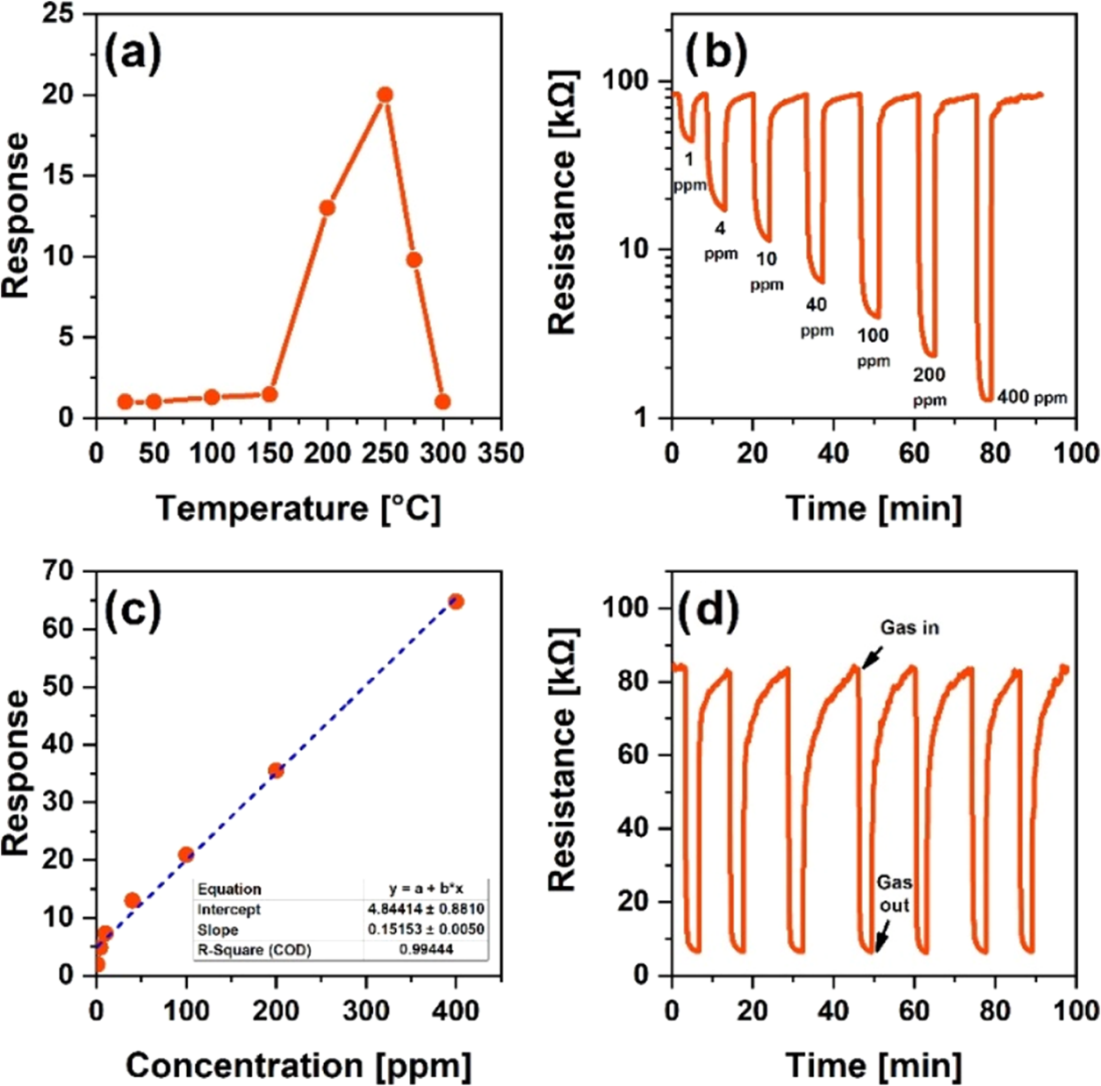
Dependence of the ethanol response on the operating temperature in the range of 25–300 °C at 40 ppm ethanol concentration (a), gas responses of ethanol toward different concentrations at 200 °C (b), dependence of the sensor’s response on ethanol vapor concentration at 200 °C (c), and response repeatability of the sensor to 40 ppm ethanol vapor expressed in seven cycles at 200 °C (d).

The SnO_2_/MWCNTs material is also demonstrated as a selective ethanol sensor using a temperature modulation method. It should be noted here that the response of the sensor to acetone in the entire temperature range (25–300 °C) was also checked by applying UV (ultraviolet) rays to the active surface. In the case of acetone vapor, temperature heating combined with UV irradiation did not produce any positive results, in contrast to the case of ethanol vapor. In this case, the simultaneous application of temperature heating and UV rays led to the acquisition of significant values of the sensor response, as shown in Figure 12a. The effect of UV radiation mainly leads to the creation of light-generated free charge carriers, which contribute to chemisorption processes, especially for alcohol sensing. Here, the maximum response was observed at 250 °C, but from the point of view of selectivity, it was appropriate to choose an operating temperature of 200 °C as an ethanol sensor.

Thus, at a working temperature of 200 °C, the characteristics of the gas-sensing parameter to the ethanol vapor were thoroughly investigated. Time-dependent curves of sensor resistance were plotted for seven different concentrations of ethanol vapor ranging from 1 to 400 ppm (Figure 12b). The justification for this choice is best reflected in Table 1.

**Table 1:** Comparison of Sensor Response Values toward 40 ppm Acetone and Ethanol at Different Operating Temperatures with and without UV Irradiation

	acetone response		ethanol response	
temperature [°C]	UV on	UV off	UV on	UV off
200	1.58	4.6	13	1
250	17	11.1	20	16
275	10.4	64.5	9.8	8.5

The dynamic resistance curves were clearly reproducible relative to the baseline resistance value, showing the independence of the concentration values. In this range of ethanol concentrations (1–400 ppm), the response values ranged from 2 to 65, respectively, exhibiting an evident linear behavior (Figure 12c). Unlike that for acetone, here linearity was observed over the entire range of ethanol concentrations with no saturation trends. This can presumably be attributed to the lighter molar mass of ethanol (46.068 g/mol) compared to that of acetone (58.08 g/mol).^65^ More mobile ethanol molecules were quickly desorbed from the surface after chemical interaction, giving other molecules the opportunity to participate in the chemisorption processes and prevent the molecular blockage of the active surface.

The repeatability of the sensor response was also checked at a pressure of 40 ppm of ethanol (Figure 12d). Despite small deviations in the resistance values at the edges of the response curves, the sensor also showed high response stability toward the ethanol vapor.

**Figure 13:**
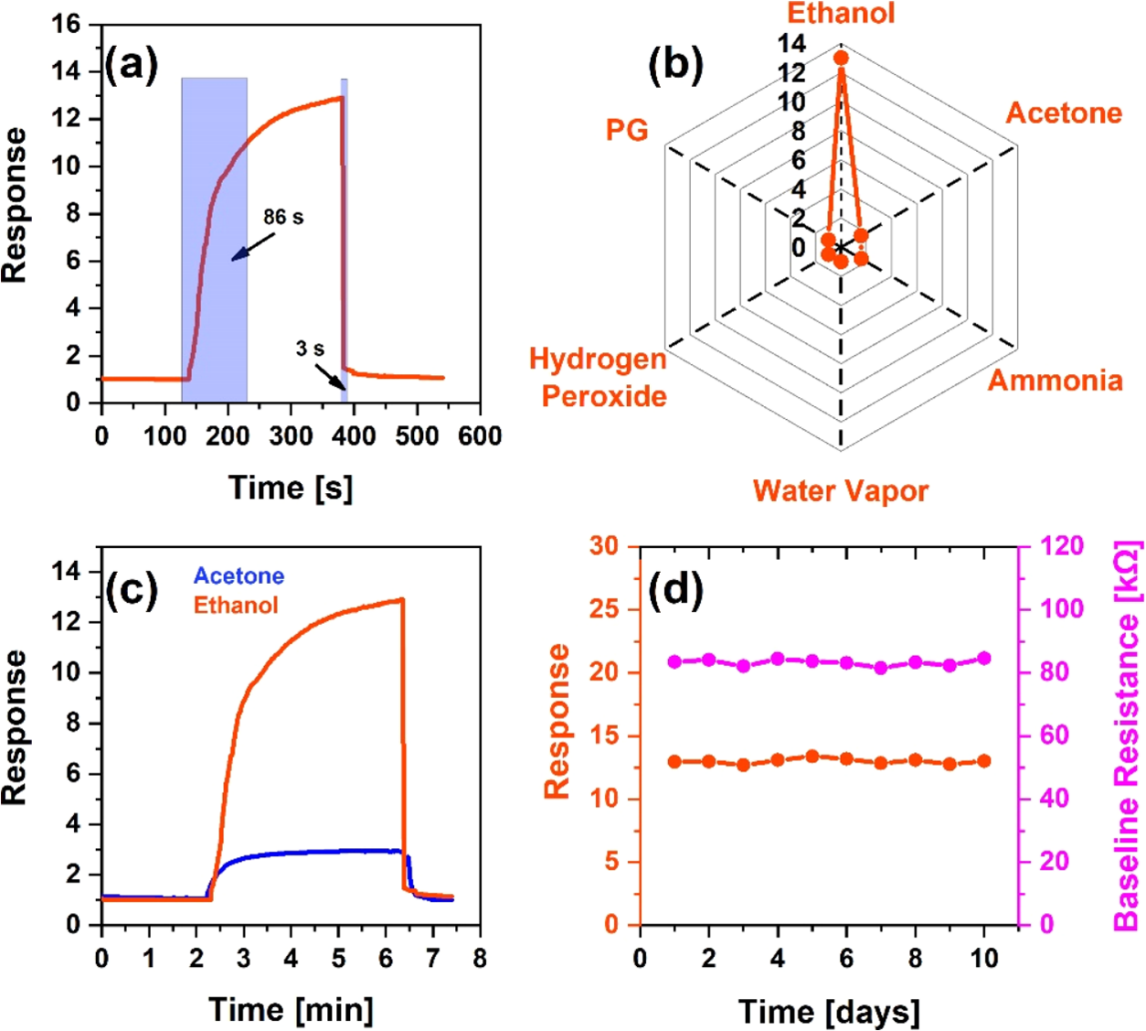
Change in the dynamic response of the sensor reflecting the response and recovery times at 40 ppm ethanol (a), ethanol selectivity of the sensor to different environmental gases (b), comparison of sensor responses to 40 ppm of ethanol and acetone vapors at 200 °C (c), and stability and baseline resistance of the sensor in 40 ppm ethanol vapor (d).

The high performance of the sensor at the operating temperature of 200 °C was confirmed by the rather short response and recovery times corresponding to the values of 86 and 3 s, respectively (Figure 13a).

The speed of the sensor in terms of recovery was extremely satisfactory, which was another indication of the high reproducibility of the sensor response. The sensor was quite selective for other surrounding gases at 200 °C (Figure 13b), where the sensor response to ethanol vapor was tens of times higher than that of all other target gases (except for acetone). Thus, the actual response curves of ethanol and acetone at 200 °C temperature were compared with the vapor concentration of 40 ppm, corresponding to values of 13 and 3, respectively (Figure 13c). In these conditions, the sensor reacted almost 4 times more than acetone, suppressing the sensor’s sensitivity to ethanol. Additionally, as a high-efficiency ethanol sensor, its long-term stability (stability for the response and baseline resistance) was also tested, which was quite satisfactory (Figure 13d).

To justify the gas-sensing results obtained for the SnO_2_/MWCNTs sensor, comparisons were made between our sensor parameters and those of other acetone sensors in the literature. Almost all parameters of our sensor were logically comparable with the characteristics of existing acetone sensors in the literature, where the extremely large response value was particularly impressive (Table 2).

**Table 2:** Summary of the Comparison between our Acetone Sensor and those Previously Fabricated and Reported in the Literature

sensing materials	operating temperature [°C]	acetone concentration [ppm]	response [*R*_air_/*R*_gas_]	refs
Pt-loaded Fe_2_O_3_ nanocubes	139	100	25.7	(66)
Au-loaded SnO_2_ nanosheets	240	100	18.2	(67)
ZnO mesoporous architectures	420	100	10.5	(68)
TiO_2_ nanorods	320	100	12.3	(69)
PdO-ZnFe_2_O_4_ macroporous spheres	350	100	18.9	(70)
PrFeO_3_ hollow nanofibers	300	100	11.6	(71)
Co_3_O_4_–SnO_2_ nanoparticles	300	50	111	(72)
SnO_2_/ZnSnO_3_ hollow microspheres	290	100	30	(73)
SnO_2_ nanofibers	280	100	32.2	(74)
Pd–NiO nanorods/SnO_2_ nanowires	450	500	14.88	(75)
SnO_2_–NiO composites (nanoparticles)	350	10	7.4	(76)
MWCNTs/SnO_2_ nanocomposite	250	100	13.5	(55)
SnO_2_/MWCNTs nanocomposite	275	100	90.5	this work
SnO_2_/MWCNTs nanocomposite	275	1	1.2	this work

Since the main focus of the article was on acetone detection, no comparison table with the characteristics of the ethanol sensor was assembled. However, the ethanol characteristics obtained by us were comparable to those of others.^77−83^

### Gas-Sensing Mechanism

**Figure 14:**
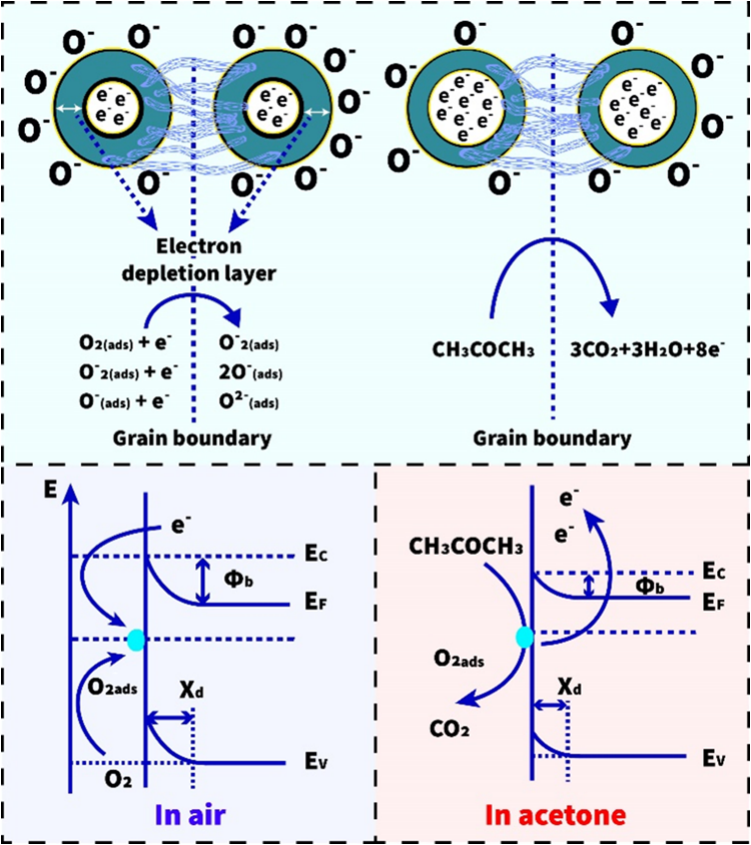
Schematic diagram of the acetone sensing mechanism for the SnO_2_/ MWCNTs material.

The gas-sensing mechanism of the SnO_2_/MWCNTs sensor can be characterized by the adsorption and desorption phenomena occurring between the target gas and the semiconductor nanocomposite. Here, the processes are more illustratively presented by the grain-boundary model, where the gas-sensitive matrix is characterized as a unique network of nanoparticles.^18,84,85^ As a result of the chemisorption of oxygen species under atmospheric conditions, depletion layers are formed in the near-surface areas of the grains, due to which electron transfer between neighboring grains is limited by the Schottky barriers formed (Figure 14).

Acetone gas molecules usually tend to react with preadsorbed oxygen ions, practically reducing their concentration on the grain surface. This steadily reduces the thicknesses of the depleted layers and the heights of the resulting Schottky barrier, leading to a sharp drop in the electrical resistance of the gas-sensitive matrix.^86−90^ Here, carbon nanotubes presumably represent unique bridges between neighboring grains through which the high-mobility electrons can pass. This leads to an increase in the sensor speed while the nanotubes’ superior ability to absorb rather more gas molecules ensures a high acetone response.^55,91^ Carbon nanotubes can also create additional sublevels within the band gap of tin oxide near the conductance and valence band edges, which would result in an effective (smaller) band gap energy. As a result, additional electron/hole pairs are generated at lower temperatures, which participate in chemisorption processes, improving the acetone sensing properties and decreasing the operating temperature.^55^ Besides, if we attribute p-type semiconductor properties to MWCNT, then it can form depletion layers with n-type SnO_2_. The expansion of the depletion layer width in the region near the surface of SnO_2_ grains implies an increase in gas response.^92,93^

## Conclusions

In summary, we applied a straightforward hydrothermal method to synthesize nanostructured SnO_2_/MWCNTs materials for the detection of VOCs. Here, SEM and TEM characterizations revealed the presence of carbon nanotubes surrounding tin oxide nanoparticles with 20–300 nm sizes. Well-expressed peaks of carbon nanotubes and tin oxide were present in the EDX and XRD spectra of the SnO_2_/MWCNTs material. Application of UV irradiation combined with the modulation of the temperature resulted in selective ethanol detection, replacing the operating temperature of 275 °C for acetone with that of 200 °C for ethanol. Among the results of the gas-sensing measurements, it was notable that the concentration range of 1–400 ppm acetone and alcohol corresponded to the ranges of response values 1.2–118 and 2–65, respectively. The high repeatability of the sensor response combined with high selectivity and temporal stability was also demonstrated. The enhanced response of the sensor was attributable to the positive change in the morphological, structural, and compositional characteristics caused by the import of the MWCNTs.
